# Detection of Exosomal microRNA from Malignant Lung Cells Using a Surface Plasmon Resonance Imaging Biosensor

**DOI:** 10.3390/bios15030159

**Published:** 2025-03-03

**Authors:** Razvan Bocu

**Affiliations:** Department of Mathematics and Computer Science, Transilvania University of Brasov, 500036 Brasov, Romania; razvan.bocu@unitbv.ro; Tel.: +40-732011010

**Keywords:** biosensors, bioimaging, malignant lung cells, NSCLC, surface plasmon resonance imaging

## Abstract

The consideration of micro ribonucleic acid (microRNA) molecules is justified by their ability to act as possible biomarkers, which may be used to detect the most prevalent type of lung cancer: non-small-cell lung cancer (NSCLC). This article describes a surface plasmon resonance imaging (SPRi) biosensor, which was considered to detect malignant lung cells using an Au/Ag heterostructure, as well as the DNA tetrahedron framework (DNATF). Considering the Au/Ag heterostructure and the DNATF, the proposed SPRi biosensor was evaluated. Thus, its detection range belongs to the interval 1.82 fM up to 28 nM. The measured limit of detection of this biosensor is 1.55 fM, and the generated images offer sufficient fidelity to conduct a precise medical analysis. Therefore, patients who suffer from NSCLC may be accurately and efficiently determined. The experimental evaluation, which is presented, suggests that this is a biosensor that is capable of optimizing the clinical detection of NSCLC.

## 1. Introduction

Considering the variety of cancer types, the lungs represent the most affected internal organs. Consequently, non-small-cell lung cancer (NSCLC) is the most frequent type of malignant disease located at the level of the lungs. Therefore, the proper detection of NSCLC malignant cells appears as a natural goal for the precise and timely detection of this problematic medical condition. Unfortunately, most clinical exams identify late stages of the disease, which determines a low survival rate of 15% over a five-year period [1,2]. Certain articles, such as [3], demonstrated that early medical examinations may improve the prognostic, and also diminish the overall cost of the medical procedures. Nevertheless, regular investigation methods, which consider the biopsy of the tissues, proteomic biomarkers, or in vivo imaging exhibit a low throughput of the detection process, and a related low accuracy of the overall detection process. Consequently, it is relevant to assert that the design and effective usage of accurate detection methods for NSCLC remains a stringent research topic.

The exosomes represent nanovesicles, which are secreted into the body fluids by living cells. Generally, their size ranges from 30 nm to 150 nm [4]. They are able to intimately mediate the communication that is established between cells, which involves the proper transfer of biomolecules like proteins, RNA, and DNA [5]. Thus, it is possible to identify exosomal microRNA structures, which is a type of small RNA that is endogenous and non-coding, which regulate the level of proteins through the removal of the expression of the reference microRNA molecules. This fundamentally determines the tumor growth and the subsequent metastasis [6].

Furthermore, it is relevant to note that certain papers, which we have carefully reviewed, suggested that particular exosomal microRNA structures, more precisely microRNA-21, microRNA-139, microRNA-200, and microRNA-378 represent viable biomarkers that may be used to screen NSCLC disease [7]. Moreover, it is relevant to note that exosomal microRNA structures exhibit two particular features. Thus, the concentration of exosomes, which is determined by the malignant cells, is substantially greater than the related concentration of exosomes generated by the healthy cells [8]. Additionally, the membrane of the exosomes preserves the included microRNA molecules from possible degradation through interaction with Ribonuclease, which favors breaking RNA into smaller particles [9]. Therefore, the detection of exosomal microRNA molecules appears as a very promising approach for the detection and precise diagnosis of the NSCLC disease.

MicroRNA molecules can be analyzed using several methods. One of the approaches is designated Quantitative Reverse Transcription Polymerase Chain Reaction (QRT-PCR) [10]. This approach was experimentally discovered to generate a sufficiently high rate of false positive errors. This issue is determined by the defining features of the microRNA molecules, especially high sequence similarity and short length. Therefore, the design and development of alternative detection models for microRNA molecules constitute an important research topic, which is discussed in certain papers, such as [11,12]. The main disadvantage of these solutions is represented by the laborious labeling of the molecules that generate signals, such as ferrocene and methylene blue. Additionally, it is difficult to extract fully conclusive information from the analysis of single microRNA molecules, considering that a specific biomarker may relate to several types of cancer. Therefore, a precise detection of NSCLC malignant cells requires the detection of several microRNA molecular samples relative to a particular clinical trial.

In this context, it may be stated that surface plasmon resonance imaging (SPRi) biosensors represent a high-throughput system, which is suitable for the detection of clinical analytes in a real-time manner [13]. Relative to other sensing systems, such as fluorescence or electrochemistry, SPRi biosensors do not need special reagents, or special dyes in order to generate output signals [14]. Considering these features, the related biosensor represents an efficient biosensing system, which may be considered to simultaneously detect multiple exosomal microRNA molecules. Nevertheless, it remains a difficult task to detect the indications of exosomal microRNA molecules through the utilization of SPRi biosensors, considering that an efficient surface plasmon resonance (SPR) amplifier cannot be properly determined. Conversely, the non-standard adsorption of convoluted biological patterns, such as nucleic acids, proteins, and lipids on the target sensors and chips, may obscure particular concrete signals, which would produce a significant reduction in the assay’s sensitivity and specificity. This may effectively rule out this technical solution’s relevance as a fully featured and precise clinical solution [15].

There are certain metal structures, such as silver (Ag) and gold (Au), which may be considered to improve the strength of the SPR biosensors [16]. Nevertheless, individual metal nanostructures are experimentally proven to be limited in terms of sensitivity. Therefore, there are certain biotechnological approaches that introduce hybrid metal nanostructures [16,17]. The direct and simple assembly of hybrid metal nanostructures may consider DNA molecules as functional linking structures [18]. Thus, relevant solutions relate to a combination of single-stranded DNA (SSDNA) and additional sequences that are placed on the metal nanostructure [19]. Therefore, the hybrid metal nanostructures may be considered to design and implement the novel structures that are necessary to augment the power of the SPR signals.

The approach, which is presented in this paper, is featured by certain defining novel features. Therefore, this paper presents an SPRi biosensor that may be considered to accurately detect malignant lung cells, which are related to the NSCLC type of cancer. The hybrid implementation model of this biosensor considers an Au/Ag structure, and the DNA tetrahedron framework (DNATF). The selection of DNATF is justified by its structural stability and mechanical stiffness [20]. The inclusion of DNATF enhances the probes’ binding efficiency, and it also improves the antifouling capabilities of the biosensor’s interface [21].

Thus, exosomal microRNA molecules attached to the DNATF probes and affixed to a gold array chip favor the self-assembly properties of the Au/Ag hybrid metal nanostructures. This solution determines an enhancement of the signal that sustains the precise detection of the exosomal microRNA molecules. The resulting high-throughput biosensor constitutes an adequate solution for the detection of the target malignant cells and consequently the timely diagnosis of NSCLC.

The rest of this paper is structured according to the following sections: The materials and methods are thoroughly presented. Next, the scientific results are discussed, and the outcomes of the experimental evaluation process are analyzed. The last section concludes the paper.

## 2. Materials and Methods

The experimental process that is described was organized in the laboratory that was provided by the partner biomedical company.

### 2.1. Basic Materials and Chemical Compounds

The initial stages of the biosensor design, as well as the experimental process, considered certain materials and chemical compounds. The partner biomedical company communicated the following details. Sodium hydrosulfide hydrate (NaSH), ethylene glycol (EG), and silver trifluoroacetate (${\mathrm{CF}}_{3}\mathrm{COOAg}$), were sourced from from Sigma-Aldrich Chemical Co., Ltd. (St. Louis, MO, USA). The bovine serum albumin (BSA) was purchased from Thermo Fisher Scientific Co., Ltd. (Wilmington, NC, USA). Additionally, it is relevant to note that the liquid compounds were based on ultrapure water offered by Merck Millipore from Arklow, Wicklow, Ireland. Naturally, the other necessary reagents were featured by a purity of at least 95%, which ensures they were proper for the implied analytical processes.

### 2.2. Considered Devices and Related Measurements

The initial stages of the experimental process considered the design and development of an SPRi biosensor, which was considered to evaluate the exosomal microRNA molecules. The SPRi biosensor is made of a laser component, which is used to excite the SPR components. The biosensor assembly also includes a CCD camera, which has the role of collecting the required biological snapshots, and a sensor chip. This setup is capable of detecting multiplex target entities considering three channels. Additionally, a diode that emits a red light with a wavelength of 655 nm was included in the configuration.

The P-polarization of the light was generated through a polarizing sheet. The matching liquid compound was processed between the prism and the sensor chip. The generated image was reflected through the prism, and it was collected using a QImaging Retiga 1300 CCD camera (Burnaby, BC, Canada), which supports a 12-bit color depth. The sensorgrams, which were obtained using the biosensor assembly, were evaluated through a software application that was implemented in LabVIEW 2023, a platform that is developed by National Instruments. The obtained sensorgrams were used to create a chronological flow of the molecular interactions, and the response was measured in Resonance Units (RU). It is relevant to note that the angle of the SPR is determined by the RU. Thus, 1000 RU generates an approximate angle variation of 0.1°. It is interesting to note that a positive correlation between the variation in the angle and the mass of the biosensor’s surface was experimentally determined. Therefore, the implemented SPRi biosensor detected the angular variation by measuring the intensity of the reflected light, considering a fixed angle of incidence.

### 2.3. Generation of the DNATF Probes

The generation of the DNATF probes conforms to the technique that is referred to as base pairing. Further interesting details were reported in the article in [22]. Thus, the process begins with the dissolution of the DNA strands using TM Buffer produced by Rockland in Philadelphia, PA, USA. The TM Buffer solution has the following features: 50 mM Tris HCl, pH 7.5, 10 mM magnesium sulfate. Following, four DNA strands, with a size of 12 µM, were combined and kept at 95 °C for a period of seven minutes. Immediately after this step was completed, the compound was kept at 3 °C for a period of two minutes to generate the DNATF probes. It is also important to note that the obtained DNATF probes were stored at 3 °C in order to ensure their future reuse capabilities.

### 2.4. Affixing the DNATF Probes to the Gold Array Chip

Before applying the DNATF probes, the surface of the gold array chips went through a sanitization process. Thus, it was initially cleaned with a piranha solution, which was made of 70% $H_{2}{\mathrm{SO}}_{4}$, and 30% $H_{2}O_{2}$, for a period of 15 min in order to remove the existing impurities. Next, it was attentively rinsed using ultrapure water, and the chip was dried up with nitrogen. Then, the generated DNATF probes were affixed to the chip and kept at 3 °C for a period of 12 h. The DNATF probes have the following physical features: 50 µL, and 1.5 µM. The assembly of the DNATF probes on the gold chip was realized using an Au-S bonding chemical process. Furthermore, bovine serum albumin (BSA), with a concentration of 2%, and Mean Corpuscular Hemoglobin (MCH), with a concentration of 1.5% were maintained with the chip for a period of one hour, and 45 min, respectively. It is relevant to note that the functional efficiency of the chip containing DNATF probes was not affected by either BSA nor MCH. Following a supplementary cleaning stage using ultrapure water, and an attentive drying using nitrogen, the resulting chip was added to the SPRi biosensor for reuse purposes.

### 2.5. Quantitative Evaluation of the SPRi

The process begins with an injection of Phosphate-Buffered Saline (PBS) into the target device according to a speed of 10 µL min^−1^. As soon as the device attained a state of equilibrium, the intended targets were added according to a speed of 65 µL min^−1^. After three minutes and twenty seconds, the speed of the process was changed down to 15 µL min^−1^ to ensure that enough hybridization time was granted to the DNATF probes. As soon as the SPR signal became stable, the SSDNA-based silver nanoclusters (AgNC) were added to the flow cell to ensure the hybrid nature of the acquired targets. Furthermore, two types of SSDNA-based gold nanoparticles (AuNP) were consecutively added to the flow cell at the same speed in order to generate the necessary Au/Ag heterostructure. Lastly, it is important to state that PBS was added once more according to a speed of 12 µL min^−1^. This operation has the role of cleaning the chip and evaluating the binding affinity.

### 2.6. Isolation of the microRNA Molecules

This operation is determined by several steps. At the beginning, plasma was centrifuged according to a G Force of 15,000× *g* for a period of 40 min, thrice. The role of this step is to remove any existing debris, including potential large microvesicles, or entire cells. Following, the supernatant particles were acquired and centrifuged according to a G Force of 150,000× *g* for a period of 90 min, which ensures a proper collection of the exosomes. The related supernatant was poured off, and the obtained exosomes were added to a PBS solution, which was refined using 18 µm filters, and consequently centrifuged according to a G Force of 150,000× *g* for a period of 90 min. This additional step has the role of removing any remaining proteins. As soon as the supernatant was removed, the microvesicles were suspended in 150 µL PBS and kept in storage at −90 °C before their effective utilization. It is relevant to note that the centrifugation and sampling processes were conducted at a temperature of 3 °C. Additionally, the total RNA was obtained out of the exosome using Trizol, which was produced by Thermo Fisher Scientific (Waltham, MA, USA) [23].

## 3. Results and Discussion

### 3.1. Design Features of the SPRi Biosensor

**Figure 1:**
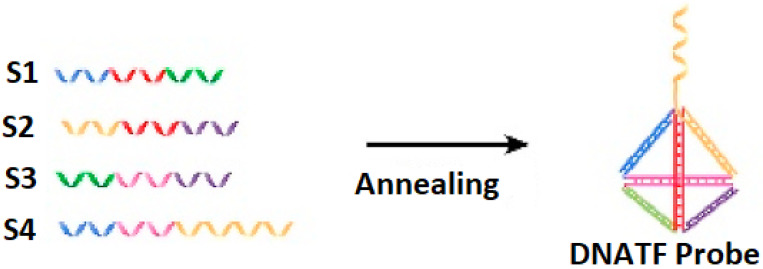
Basic hybridization mechanism of the four strands (S1, S2, S3, S4) that generate the DNATF probe.

The generation of the DNATF probes considers the hybridization mechanism, which is illustrated in Figure 1. It is relevant to note that the unrestricted sequence of the DNATF was considered to obtain the exosomal microRNA molecules.

**Figure 2:**
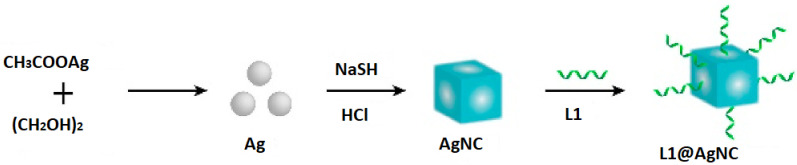
Generation mechanism of the AgNC.

Moreover, AgNC was generated and then made operational using SSDNA (L1). Consequently, this resulted in the generation of the L1@AgNC. This process is illustrated in Figure 2.

**Figure 3:**
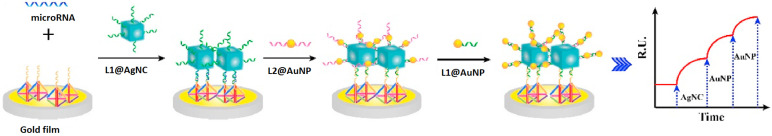
Generation of the Au/Ag structure relative to the SPRi biosensor’s chip.

Additionally, the generation of the Au/Ag structure relative to the SPRi biosensor’s chip is presented in Figure 3. Thus, a section of the target was tied to the DNATF probes affixed to the biosensor’s chip. Furthermore, the L1@AgNC compound is mixed with the remaining section of the target to generate the related compound structure. As a last step, the L1@AuNP and L2@AuNP were aggregated relative to the surface of the AgNC considering a hybridization of the strands. This step of the process effectively generated the Au/Ag structure, and it also induced the required amplification of the signal.

**Figure 4:**
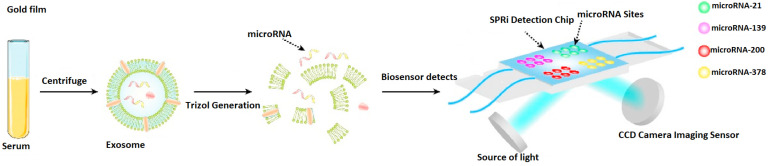
Concurrent analysis capabilities of the proposed biosensor.

It is interesting to note that, in Figure 4, the concurrent analysis capabilities of the proposed biosensor are illustrated. Thus, various DNATF probes are placed on the four detection zones of the SPRi biosensor’s chip, which allows the medical practitioner or scientific researcher to achieve a more efficient, time-sensitive evaluation of the exosomal microRNA molecules.

### 3.2. Validity of the Proposed Biosensor Assembly

**Figure 5:**
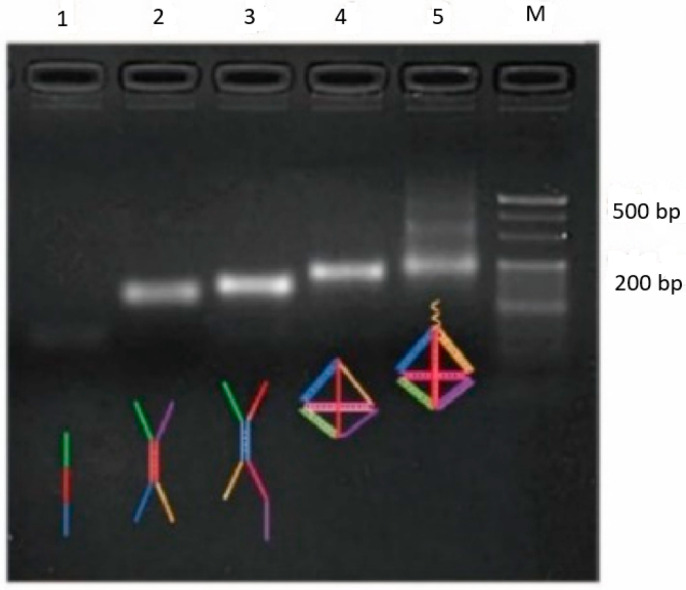
Evaluation of the DNATF probes through electrophoresis using 4% Invitrogen Agarose Gel. The concentration of the DNA strands is 1.5 µM.

The proper generation of the DNATF probes was evaluated. Thus, the shape and dimension of the generated DNATF probes were assessed through electrophoresis using 4% Invitrogen Agarose Gel from Thermo Fisher Scientific. The configuration that is presented in Figure 5 suggests and demonstrates that the DNATF probes in column 5 evolve in a slower manner than the DNATF probes in column 1 and are also relative to the DNATF probes in columns 2, 3, and 4.

**Figure 6:**
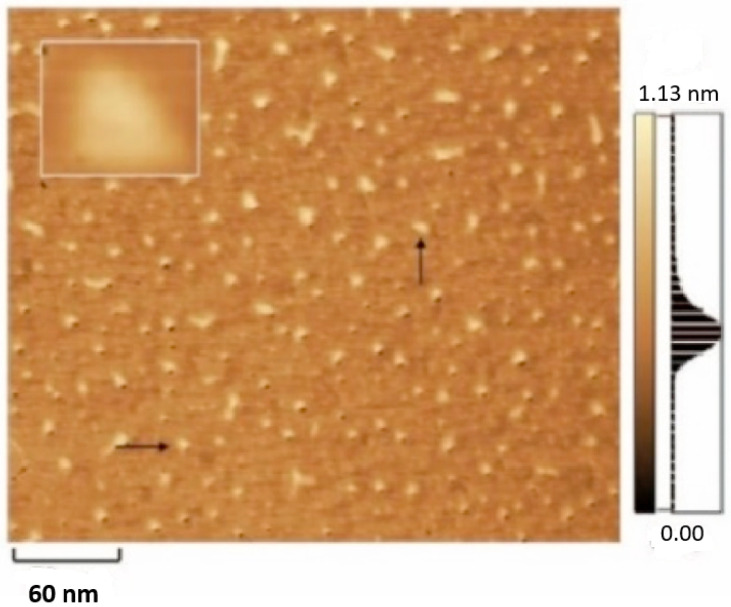
Atomic force microscopy representation of the generated DNATF probes. The considered scale bar is 60 nm.

Furthermore, the representation that is depicted in Figure 6 includes numerous tiny triangles, which is consistent with the normal structure of a proper DNATF probe. Thus, it is possible to assert that the DNATF probes were successfully generated.

Furthermore, it is relevant to state that relative to the intended microRNA molecules, the biosensor’s SPR signal progressively augmented through the inclusion of L1@AuNP, L1@AgNC, and L2@AuNP. This indicates that the gold nanoparticles (AuNP) were affixed to the surface of the silver nanoclusters (AgNC), which demonstrates the generation of the Au/Ag structure. These experimental results demonstrate that the SPRi biosensor is adequate for the analysis of exosomal microRNA molecules.

### 3.3. Assessment of the Au/Ag Structure

The experimental evaluation process also considered a comparative analysis of the Au/Ag structure, relative to AuNP and AgNC. Thus, these nanolevel structures exhibited consistency and dissipation, which proved the adequate generation of AuNP and AgNC. The obtained experimental results demonstrate that the implemented biosensor’s SPR signals vary relative to the types of metal structures that were used. Thus, the optimal results are obtained using Au/Ag, and they decreased progressively relative to AgNC and AuNP, in this order.

### 3.4. Antifouling Capability of Implemented SPRi Biosensor

The experimental evaluation thoroughly evaluated the biosensor’s antifouling capabilities. Thus, relative to the DNATF-based chip, the biosensor exhibited a reduced level of background noise, which naturally enhanced the biosensor’s detection sensitivity. The superior detection signal was registered relative to a target concentration of 35 nM. Consequently, the proposed biosensor solution may be considered to conduct the respective experimental and medical analyses, with a sufficient degree of accuracy. Additionally, it was demonstrated that the biosensor possesses the required antifouling capabilities.

### 3.5. Sensitivity of the SPRi Biosensor

**Figure 7:**
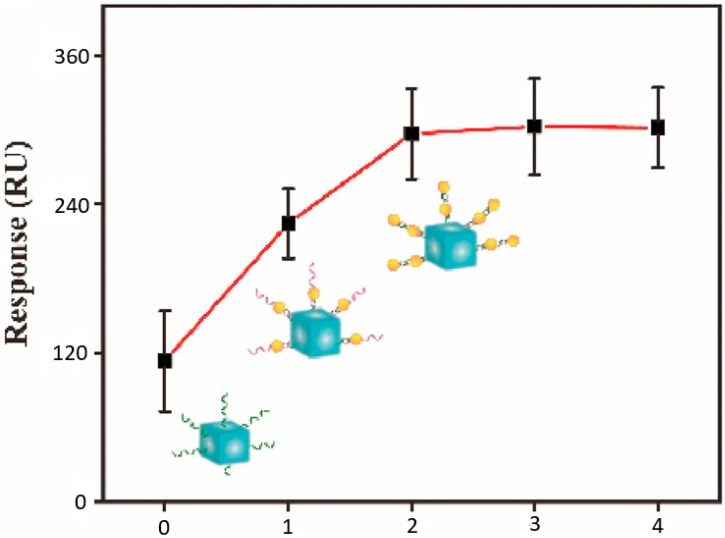
Optimization of the SPRi biosensor’s sensitivity relative to the AuNP and AgNC components. The abscissa axis represents the respective layers of the AuNP component.

The sensitivity of the implemented SPRi biosensor was gradually improved considering a step-by-step experimental calibration process. This considered incremental changes to the AuNP and AgNC components, as well as to the amount of the included DNATF probes. Thus, a summary of the AuNP and AgNC analysis is displayed in Figure 7. The biosensor’s SPR signal augmented directly proportionately to the layers of AuNP, and the optimal signal level was obtained with two layers of the AuNP component.

**Figure 8:**
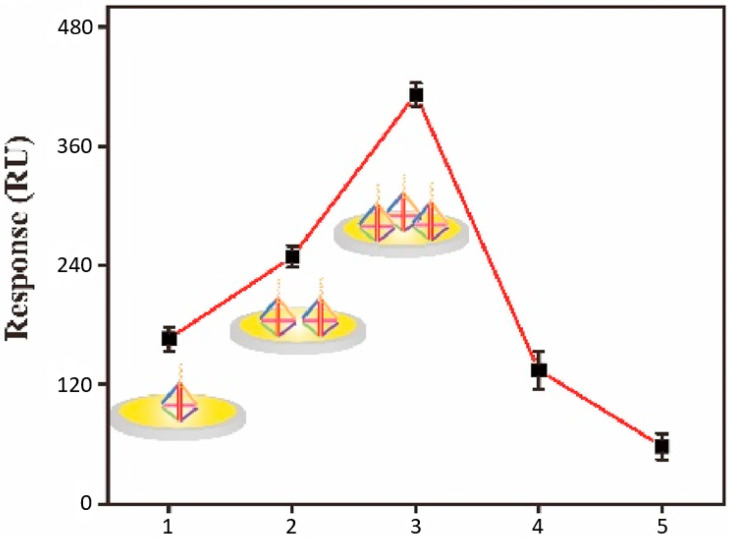
Optimization of the SPRi biosensor’s sensitivity relative to the amount of the DNATF probes. The abscissa axis represents the actual concentration of the DNATF probes, expressed in µM.

Furthermore, the amount of the DNATF probes was determined as a key factor relative to the biosensor’s detection accuracy. The results of this analysis are summarized in Figure 8. Thus, the SPR detection signal augmented directly proportional to the amount of the DNATF probes, up to a maximum level of 3 µM. Further increases in the amount of the DNATF probes reduced the detection performance of the biosensor, which would be explained by the absence from the analysis of covered experimental particles, which are part of the DNATF probes. Consequently, the optimal level of DNATF probes was validated at 3 µM.

Considering an ideal experimental scenario, the effective sensitivity of the biosensor was evaluated. Thus, the biosensor’s SPR signal is augmented relative to the target concentration according to the range 1.82 fM to 28 nM. Additionally, the limit of detection (LOD) was also considered as a performance metric for the biosensor’s detection capability. The determined value of LOD is 1.55 fM.

The proper amplification of the signal based on the utilization of AuNP is justified through a comparative analysis of the AgNC-based version of the biosensor, which produced a sensibly greater value of the LOD, at 3.29 fM. This suggestively validates the consideration of AuNP as a solution to enhance the proposed SPRi biosensor’s sensitivity. The detection performance of the proposed biosensor is further complemented and improved by the inclusion of the antifouling capabilities, which have already been discussed.

Moreover, it is relevant to note that compared with existing multiplex biosensor systems, which need to label fluorophores [24], the proposed SPRi biosensor determines more accurate detection processes generated by the lower value of the LOD. The value of LOD was calculated considering the standard procedure based on the calibration curve, which was also presented in article [24].

### 3.6. Effective Detection of microRNA Molecules

The biosensor underwent a rigorous validation process, which evaluated its capability to properly detect NSCLC malignant cells. Thus, four types of microRNA molecules were considered. These were obtained from fully anonymized exosomal clinical samples. The validation stage of the experimental process commenced with the analysis of 100 clinical instances, which included 50 healthy individuals, and 50 patients. The microRNA molecules were analyzed using Transmission Electron Microscopy (TEM). The obtained exosomal RNA molecules have the general shape of a cup, with a diameter of about 95 nm. These physical features confirm previous scientific reports, such as the one that is contained in the paper in [25].

Furthermore, the microRNA was extracted from the exosomal structures, and the related expression levels were quantified using the proposed SPRi biosensor. The experimental outcomes revealed significantly greater expression levels relative to the 50 patients’ microRNA molecules. Additionally, relative to the healthy subjects, four exosomal microRNA types of the patients exhibited overexpression. The NSCLC malignant cells were precisely identified relative to the healthy cells. Therefore, it is possible to immediately state that the proposed SPRi biosensor efficiently detects the biomarkers concerning NSCLC.

Nevertheless, we have experimentally determined that it is problematic to diagnose NSCLC relative to the exosomal variety microRNA-21. This may be due to the association of this exosomal variety with breast cancer, lung cancer, and adenocarcinoma. Consequently, it is necessary to design and implement a biosensor with multiplex microRNA detection capabilities. The proposed SPRi biosensor fully conforms to this specification. Therefore, the proposed biosensor represents an effective solution for the detection of exosomal microRNA in clinical samples.

**Figure 9:**
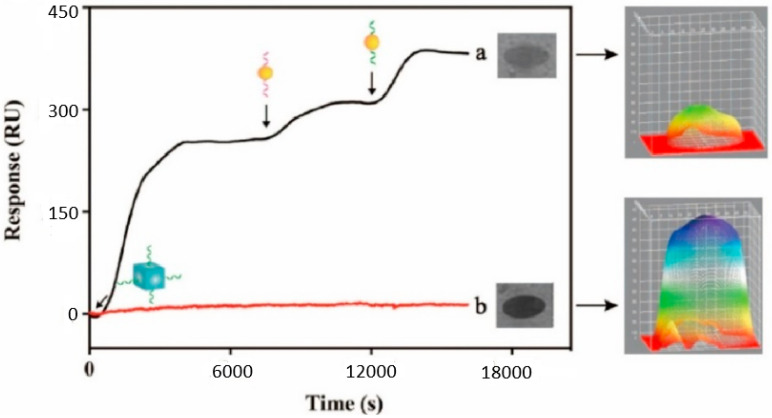
Typical SPRi-based sensorgrams related to (**a**) a 22 nM target and (**b**) a neutral sample. The evaluation of the SPRi-based 3D image is displayed on the right-hand side of the figure.

The real-world validity of the proposed approach is further demonstrated by a comparative analysis between a 22 nM sample and a neutral sample, which is represented through the sensorgrams described in Figure 9. Thus, relative to the considered microRNA molecules, the intensity of the SPRi-based signal progressively increased, relative to the successful generation of the Au/Ag structure. Conversely, if the microRNA molecules are not present on the detection chip, the SPRi-based signal exhibits a negligible variation. This further demonstrates that the proposed biosensors are adequate for the evaluation of exosomal microRNA molecules.

**Figure 10:**
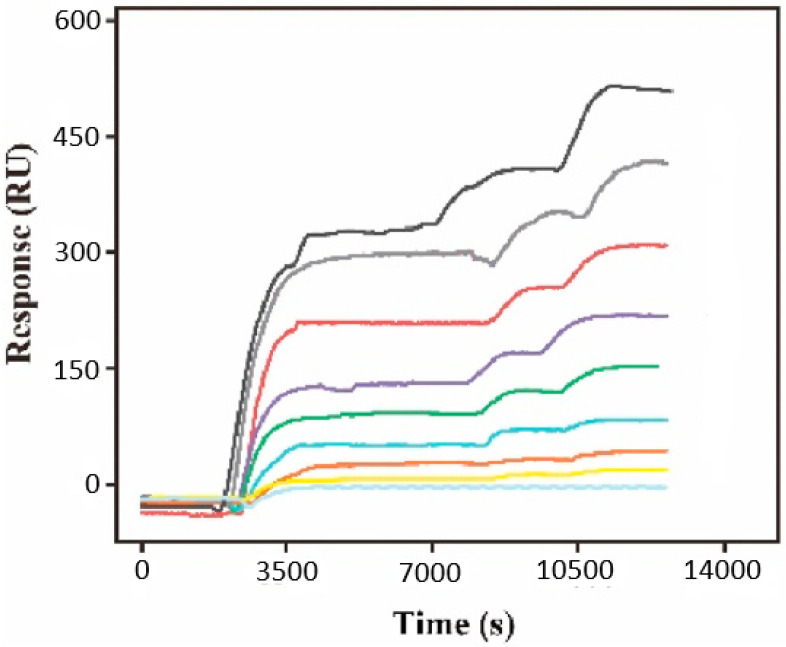
SPRi-based sensorgrams generated through the consideration of $1.5\times 10^{7}$, $1.5\times 10^{6}$, $1.5\times 10^{5}$, $1.5\times 10^{4}$, $1.5\times 10^{3}$, 150, 15, 7.5, 1.5 fM exosomal microRNA-21 molecules. The sensorgrams should be read in the downward direction, respectively.

Furthermore, it is important to note that the experimental evaluation process demonstrated an augmentation of the SPRi signal, relative to the progressive growth of the considered concentration, from 1.5 fM to 15 nM. This evolution is represented through the sensorgrams in Figure 10. This behavior also proves that the proposed biosensors are adequate for the evaluation of the considered exosomal microRNA molecules.

### 3.7. Comparative Justification of Proposed Approach Validity

It is relevant to note that three works are particularly relevant for the decision to consider the detection of microRNA molecules using an SPRi biosensor, and gold nanoparticles (AuNP).

Thus, the approach that was reported in article [26] considers the DNA walker and CHA cascade amplification, and the related detection model proved improvements of the sensitivity levels with the LOD of 42 aM. This solution is claimed to have offered experimental improvements regarding the detection of microRNA-21 detection processes.

The contribution that was reported in article [27] described a nanosystem based on AuNP, which is claimed to provide enhanced detection of microRNA molecules. This improved model can be used to set up scientific research and medical biosensing processes, which feature accurate detections of the target molecules. Another particularly interesting contribution was reported in the paper in [28], which studied miR-214, which has the potential to favor the metastasis of melanoma.

Although these reference solutions realize the detection of microRNA molecules using AuNP particles, our solution is superior in several respects. Thus, although the SPRi biosensor is optimized to detect NSCLC cells, it may be considered for the detection of other medical conditions. The detection structure exhibits a superior level of stability and mechanical stiffness, owing to the considered DNA tetrahedron framework. This solution also provides enhanced antifouling capabilities, while ultimately improving the detection signals, which effectively provides superior resolution images. This warrants this solution’s potential as a more robust NSCLC detection approach, which may be considered in both scientific research and medical real-world use case scenarios.

It is relevant to note that ultrasmall gold nanoclusters (AuNC) also represent an interesting solution for the design of molecular detection solutions. Nevertheless, as we have not been able to evaluate this variant over the course of this experimental process, it will be assessed within the frame of future scientific research projects, which will also further examine the selectivity of this approach relative to other microRNA molecules.

## 4. Conclusions

This paper presented an innovative SPRi biosensor for the accurate detection of multiplex microRNA molecules, which relates to an Au/Ag heterostructure and DNATF. The biosensor is validated relative to the detection of malignant lung cells.

The exosomal microRNA molecules are attached to the DNATF probes and also affixed to a gold array chip. This favors the self-assembly properties of the Au/Ag hybrid metal nanostructures. This solution determines an enhancement of the signal that sustains the precise detection of the exosomal microRNA molecules. The obtained high-throughput biosensor constitutes an adequate solution for the detection of the target malignant cells and consequently the timely diagnosis of NSCLC.

A comparative analysis with similar biosensors, which were reported in the scientific literature, demonstrated that this SPRi biosensor has some advantages considering the evaluation of exosomal microRNA. Thus, affixing the DNATF on the gold array chip diminishes the non-specific adsorption. This warrants the operation of the biosensor with complex fluids. Furthermore, considering the inclusion of the Au/Ag as a design solution, this biosensor ensures a high level of sensitivity, at 1.55 fM.

It is also relevant to note that the biosensor possesses concurrent (multiplex) detection capabilities, which allows it to discover four microRNA varieties in a single clinical sample. These features imply that the biosensor is able to efficiently detect the biomarkers, which are related to NSCLC, while observing reliable antifouling capabilities, and high detection sensitivity. Therefore, the evaluated SPRi biosensor represents a viable solution, which can be used in various clinical scenarios. Considering the versatility of this approach, we plan to further improve it in the realm of our continued collaboration with the partner biomedical engineering company. 

## Data Availability

No new data were created or analyzed in this study. Data sharing is not applicable to this article.

## References

[1] Siegel R.L., Miller K.D., Fedewa S.A., Ahnen D.J., Meester R.G., Barzi A., Jemal A. (2017). Colorectal cancer statistics. CA Cancer J. Clin..

[2] Pao W., Girard N. (2011). New driver mutations in non-small-cell lung cancer. Lancet Oncol..

[3] Rabinowits G., Gerçel-Taylor C., Day J.M., Taylor D.D., Kloecker G.H. (2009). Exosomal microRNA: A diagnostic marker for lung cancer. Clin. Lung Cancer.

[4] Zomer A., Vendrig T., Hopmans E.S., van Eijndhoven M., Middeldorp J.M., Pegtel D.M. (2010). Exosomes: Fit to deliver small RNA. Commun. Integr. Biol..

[5] Mathieu M., Martin-Jaular L., Lavieu G., Théry C. (2019). Specificities of secretion and uptake of exosomes and other extracellular vesicles for cell-to-cell communication. Nat. Cell Biol..

[6] Jiang Y., Shi M., Liu Y., Wan S., Cui C., Zhang L., Tan W. (2017). Aptamer/AuNP biosensor for colorimetric profiling of exosomal proteins. Angew. Chem. Int. Ed..

[7] Cazzoli R., Buttitta F., Nicola M.D., Malatesta S., Marchetti A., Rom W.N., Pass H.I. (2013). microRNAs derived from circulating exosomes as noninvasive biomarkers for screening and diagnosing lung cancer. J. Thorac. Oncol..

[8] Heneghan H.M., Miller N., Kelly R., Newell J., Kerin M.J. (2010). Systemic miRNA-195 differentiates breast cancer from other malignancies and is a potential biomarker for detecting noninvasive and early stage disease. Oncologist.

[9] Liu L., Lu H., Shi R., Peng X.-X., Xiang Q., Wang B., Wan Q.-Q., Sun Y., Yang F., Zhang G.-J. (2019). Synergy of peptide–nucleic acid and spherical nucleic acid enabled quantitative and specific detection of tumor exosomal MicroRNA. Anal. Chem..

[10] Tavallaie R., McCarroll J., Grand M.L., Ariotti N., Schuhmann W., Bakker E., Tilley R.D., Hibbert D.B., Kavallaris M., Gooding J.J. (2018). Nucleic acid hybridization on an electrically reconfigurable network of gold-coated magnetic nanoparticles enables microRNA detection in blood. Nat. Nanotechnol..

[11] Ma D., Huang C., Zheng J., Tang J., Li J., Yang J., Yang R. (2018). Quantitative detection of exosomal microRNA extracted from human blood based on surface-enhanced Raman scattering. Biosens. Bioelectron..

[12] Xia Y., Wang L., Li J., Chen X., Lan J., Yan A., Lei Y., Sheng Y., Huanghao Y., Chen J. (2018). A ratiometric fluorescent bioprobe based on carbon dots and acridone derivate for signal amplification detection exosomal microRNA. Anal. Chem..

[13] Mubarak Z.H.A., Premaratne G., Dharmaratne A., Mohammadparast F., Andiappan M., Krishnan S. (2020). Plasmonic nucleotide hybridization chip for attomolar detection: Localized gold and tagged core/shell nanomaterials. Lab. Chip..

[14] Wang Q., Zou L., Yang X., Liu X., Nie W., Zheng Y., Cheng Q., Wang K. (2019). Direct quantification of cancerous exosomes via surface plasmon resonance with dual gold nanoparticle-assisted signal amplification. Biosens. Bioelectron..

[15] Jiang C., Wang G., Hein R., Liu N., Luo X., Davis J.J. (2020). Antifouling strategies for selective in vitro and in vivo sensing. Chem. Rev..

[16] Zhang Y., Shuai Z., Zhou H., Luo Z., Liu B., Zhang Y., Zhang L., Chen S., Chao J., Weng L. (2018). Single-Molecule Analysis of MicroRNA and Logic Operations Using a Smart Plasmonic Nanobiosensor. J. Am. Chem. Soc..

[17] Lambert A., Yang Z., Cheng W., Lu Z., Liu Y., Cheng Q. (2018). Ultrasensitive Detection of Bacterial Protein Toxins on Patterned Microarray via Surface Plasmon Resonance Imaging with Signal Amplification by Conjugate Nanoparticle Clusters. ACS Sens..

[18] Laramy C.R., O’Brien M.N., Mirkin C.A. (2020). Crystal engineering with DNA. Spherical Nucleic Acids.

[19] Wang Y., Zhao Y., Bollas A., Wang Y., Au K.F. (2021). Nanopore sequencing technology, bioinformatics and applications. Nat. Biotechnol..

[20] Xu S., Chang Y., Wu Z., Li Y., Yuan R., Chai Y. (2020). One DNA circle capture probe with multiple target recognition domains for simultaneous electrochemical detection of miRNA-21 and miRNA-155. Biosens. Bioelectron..

[21] Nie W., Wang Q., Zou L., Zheng Y., Zheng Y., Liu X., Yang X., Wang K. (2018). Low-Fouling Surface Plasmon Resonance Sensor for Highly Sensitive Detection of MicroRNA in a Complex Matrix Based on the DNA Tetrahedron. Anal. Chem..

[22] Lin M., Song P., Zhou G., Zuo X., Aldalbahi A., Lou X., Shi J., Fan C. (2016). Electrochemical detection of nucleic acids, proteins, small molecules and cells using a DNA-nanostructure-based universal biosensing platform. Nat. Protoc..

[23] Lässer C. (2013). Identification and analysis of circulating exosomal microRNA in human body fluids. Circulating MicroRNAs: Methods and Protocols.

[24] Wang H., He D., Wan K., Sheng X., Cheng H., Huang J., Zhou X., He X., Wang K. (2020). In situ multiplex detection of serum exosomal microRNAs using an all-in-one biosensor for breast cancer diagnosis. Analyst.

[25] Tang Y.T., Huang Y.Y., Zheng L., Qin S.H., Xu X.P., An T.X., Xu Y., Wu Y.S., Hu X.M., Ping B.H. (2017). Comparison of isolation methods of exosomes and exosomal RNA from cell culture medium and serum. Int. J. Mol. Med..

[26] Li J., Li H.W. (2023). Ultrasensitive miRNA Detection by AuNP-based 3D DNA Walker and Catalytic Hairpin Assembly (CHA) Cascade Amplification for Early Cancer Diagnosis. Chemistry.

[27] Dong C., Xiong J., Ni J., Fang X., Zhang J., Zhu D., Weng L., Zhang Y., Song C., Wang L. (2022). Intracellular miRNA-triggered surface-enhanced Raman scattering imaging and dual gene-silencing therapy of cancer cell. Anal. Chem..

[28] der Ven C.F.V., Tibbitt M.W., Conde J., van Mil A., Hjortnaes J., Doevendans P.A., Sluijter J.P.G., Aikawa E., Langer R.S. (2021). Controlled delivery of gold nanoparticle-coupled miRNA therapeutics via an injectable self-healing hydrogel. Nanoscale.

